# Performance of a Three-Tier (IRT-DNA-IRT) Cystic Fibrosis Screening Algorithm in British Columbia

**DOI:** 10.3390/ijns6020046

**Published:** 2020-06-02

**Authors:** Graham Sinclair, Vanessa McMahon, Amy Schellenberg, Tanya N. Nelson, Mark Chilvers, Hilary Vallance

**Affiliations:** 1 Department of Pathology and Laboratory Medicine, BC Children’s Hospital, University of British Columbia, Vancouver, BC V6H 3N1, Canada; tnelson@cw.bc.ca (T.N.N.); hvallance@cw.bc.ca (H.V.); 2 Department of Pediatrics, BC Children’s Hospital, Vancouver, BC V6H 3N1, Canada; vmcmahon@cw.bc.ca (V.M.); amy.schellenberg@viha.ca (A.S.); 3 Department of Pediatrics, BC Children’s Hospital, University of British Columbia, Vancouver, BC V6H 3N1, Canada; mchilvers@cw.bc.ca

**Keywords:** cystic fibrosis, newborn screening, immunoreactive trypsinogen, British Columbia, sweat test, false negative, CFTR

## Abstract

Newborn screening for Cystic Fibrosis has been implemented in most programs worldwide, but the approach used varies, including combinations of immunoreactive trypsinogen (IRT) and CFTR mutation analysis on one or more specimens. The British Columbia (BC) newborn screening program tests ~45,000 infants per year in BC and the Yukon Territory, covering almost 1.5 million km^2^ in western Canada. CF screening was initiated using an IRT-DNA-IRT approach with a second bloodspot card at 21 days of age for all CFTR mutation heterozygotes and any non-carriers in the top 0.1% for IRT. This second IRT was implemented to avoid sweat testing of infants without persistent hypertrypsinemia, reducing the burden of travel for families. Over nine years (2010–2018), 401,977 infants were screened and CF was confirmed in 76, and a further 28 were deemed CF screen positive inconclusive diagnosis (CFSPID). Day 21 IRT was normal in 880 CFTR mutation carriers who were quoted a very low CF risk and offered optional sweat testing. Only 13% of families opted for sweat testing and a total of 1036 sweat tests were avoided. There were six false negative CF cases (and three CFSPID) due to a low initial IRT or no CFTR mutations. Although one CFSPID case had a normal repeat IRT result, the addition of the day 21 IRT did not contribute to any CF false negatives.

## 1. Introduction

Cystic fibrosis (CF) is one of the few disorders for which randomized controlled trials have been conducted to provide support for population-wide newborn screening. Despite the evidence for screening and wide implementation around the world, there remains significant variability in the approach to screening between jurisdictions. While the vast majority of programs start with the measurement of immunoreactive trypsinogen (IRT) using dried bloodspots and finish with sweat chloride levels (sweat test) as a confirmatory diagnostic test, a number of secondary biochemical or molecular tests may be employed in variable sequence between these end points. Much of this diversity stems from the fact that no one test is sufficiently sensitive nor specific enough to differentiate newborns who are likely to develop clinically significant disease from the general population. The primary IRT marker by itself has a positive predictive value (PPV) for CF of less than 1% and will miss ~2–5% of cases regardless of the subsequent screening algorithm [1,2].

Despite these constraints, a number of well-designed screening algorithms have been shown to provide acceptable performance for the detection of CF in the newborn period. These algorithms, combining IRT testing and Cystic Fibrosis Transmembrane Conductance Regulator (CFTR) mutation panels (IRT-DNA, IRT-IRT, IRT-IRT-DNA, and IRT-DNA-IRT), all represent compromises with respect to cost, carrier detection, sweat testing rates, and the likelihood of identifying CFTR variants of unclear clinical significance [3,4,5,6]. Generally referred to as “CF-related metabolic syndrome (CRMS)” in the US and “CF Screen Positive Inconclusive Diagnosis (CFSPID)” in Canada and Europe, these classifications describe infants with a positive CF newborn screen and either (1) a sweat chloride value < 30mmol/L and two CFTR mutations, at least one of which has unclear phenotypic consequences, or (2) an intermediate sweat chloride value (30–59 mmol/L) and one or zero CF-causing mutations [7,8]. Such cases are a challenge for screening programs as their identification is a by-product rather than a goal of the screening program. However, 5–15% of infants initially classified as CRMS/CFSPID may go on to develop CF over time [9].

**Figure 1:**
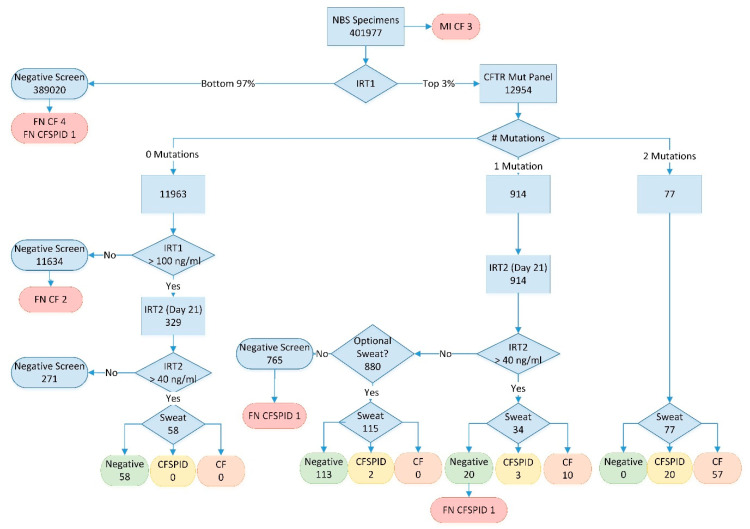
BC Cystic Fibrosis workflow 2009–2018; numbers represent total samples at each point in the algorithm over the full study period. MI = meconium ileus; IRT1 = initial IRT analysis at 24–48 h; IRT2 = repeat IRT analysis at day 21; FN = false negative; Sweat = sweat chloride testing; CF = cases meeting cystic fibrosis diagnosis criteria (see text); CFSPID = cases meeting cystic fibrosis screen positive inconclusive diagnosis criteria (see text).

The British Columbia newborn screening program introduced a three-tier screening algorithm (IRT-DNA-IRT) for Cystic Fibrosis, using a second IRT measurement at three weeks of age for all individuals with one mutation on CFTR testing or an initial IRT in the top 0.1% and no CFTR mutations, screening over 400,000 infants from 2010–2018 (Figure 1). The primary goal of this approach was to further reduce the residual risk of disease for apparent CF carriers and includes a novel optional sweat test arm for CFTR mutation carriers without persistent hypertrypsinemia. Given the wide geography covered by the screening program, including both the province of BC and the Yukon Territory (~1.5 million km^2^), but centralized sweat testing at a single center in Vancouver, this algorithm was designed to minimize the number of families required to travel for confirmatory sweat testing.

## 2. Materials and Methods

### 2.1. Data Sources

Analytical data for all infants screened in British Columbia and the Yukon from January 2010 to December 2018 (inclusive) was extracted from the Newborn Screening laboratory information system. Diagnostic testing results were obtained from the BC Children’s Hospital Cystic Fibrosis clinic records, as they maintain follow-up records for all screen-positive infants. Research ethics approval was not required for this program evaluation and quality assurance activity. Case definitions for CF and CFSPID were based on CF Foundation Consensus Guidelines [7]. Cystic Fibrosis is defined as one or two CF-causing mutations (based on CFTR2 definitions as of Jan 2019, www.cftr2.org) and a sweat chloride > 60 mmol/L. CFSPID cases are those with a positive newborn screen for CF and either (1) a sweat chloride value < 30 mmol/L and two CFTR mutations, at least one of which has unclear phenotypic consequences, or (2) an intermediate sweat chloride value (30–59 mmol/L) and one or zero CF-causing mutations.

### 2.2. Screening Algorithm

All infants in BC and the Yukon were offered newborn screening as a standard of care. Families could opt out of screening for any reason but participation rates were high for this publicly funded program. Specimens were collected by heel prick either in hospital or at home with >90% of samples collected between 24–48 h after birth. Samples were shipped to the newborn screening laboratory at BC Children’s Hospital in Vancouver for analysis. IRT testing was completed using the Perkin Elmer NIRT Autodelfia kit (Perkin Elmer Canada, Woodbridge, ON, Canada) CFTR analysis was completed in the Division of Genome Diagnostics at BC Children’s Hospital using three different methodologies across the study period due to the successive discontinuation of kits by the manufacturers. From 1 January 2010–14 July 2010, a 24-mutation panel was tested using the Signature CF 2.0 ASR assay (Asuragen Inc., Austin, TX, USA), after which testing was transitioned to a 38-mutation panel using the InPlex CF Molecular Test (Third Wave Technologies Inc./Hologic, Madison, WI, USA). Finally, in October 2016, the method was moved to a 130-mutation panel using the MiSeqDX CF 139-Variant Assay (Illumina Inc., San Diego, CA, USA). A full list of tested mutations is available at www.newbornscreeningbc.ca (see Table S1). A team of CF liaison nurses handled all communications with families and primary care providers, acting as a single point of contact and providing continuity of care throughout the screening and diagnostic follow-up process.

The CF screening algorithm is outlined in Figure 1. All infants were tested for IRT with the daily top 3% sent for CFTR analysis (performed weekly). Infants with no mutations were reported as a negative screen for CF unless their initial IRT result was in the top 0.1%. A second bloodspot card for IRT analysis at 21 days of age was requested for infants in the top 0.1% as a “fail-safe” arm. For infants with one CFTR mutation, an inconclusive result was reported and a second bloodspot card requested to be collected at 21 days of age. Finally, those infants with two CFTR mutations were reported as screen positive for CF and referred to the CF clinic for follow-up and confirmatory sweat testing.

All repeat samples (fail-safe arm and apparent CFTR carriers) with a 21-day IRT value above 40 ng/mL (90th percentile) were considered high risk for CF and the infants were referred to the CF clinic for sweat testing and clinical follow-up. Those infants without persistent hypertrypsinemia were quoted a very low (<1%) residual risk of CF and were provided with the option of coming to Vancouver for a sweat test to conclusively rule out the disease.

## 3. Results

A breakdown of the screening results are presented with the algorithm in Figure 1. Between 2010 and 2018, a total of 401,977 infants were screened with 104 cases detected (CF + CFSPID) and, of those, 76 met the definition for CF. Three infants with CF were identified prior to screening due to meconium ileus. As summarized in Table 1, most infants with CF or CFSPID were detected due to the presence of 2 mutations on CFTR analysis (76% of CF cases). Another 13% of CF cases (12.5% of CF + CFSPID) had one mutation on CFTR analysis but were shown to have persistent hypertrypsinemia on the second IRT sample (IRT2) at 21 days of age. No cases of CF or CFSPID were detected in the population of infants with no CFTR mutations but a very high initial IRT (fail-safe arm). The positive predictive value for CF with one mutation and persistent hypertrypsinemia was 29% (38% for CF + CFSPID).

With a normal IRT2 result on day 21, 880 apparent CFTR mutation carriers were given the option of a sweat test and 115 families (13%) chose to come to Vancouver for testing. No CF cases were detected by the optional sweat test; however, two infants had a borderline sweat result and are being followed as CFSPID. Including the 271 infants with normal day 21 IRT results from the fail-safe arm, a total of 1036 sweat tests were avoided by the inclusion of this second IRT, with no CF cases known to be missed as a result of this algorithm. The negative predictive value (NPV) of a normal day 21 IRT for CF mutation carriers was 100% (NPV = 99.7% for CFSPID).

There were a total of nine false negative screens in the study period, including six CF cases and three infants with CFSPID (Table 1). This amounts to an overall false negative rate of 8.8% (7.8% for CF only). The majority of missed cases (5/9) were due to normal IRT values on the initial bloodspot card. Two infants with CF were homozygous for rare mutations not on the CFTR panel but one of the two would have been detected had the current 130 mutations been in use at the time of screening. Overall, the increasing number of CFTR mutations tested did have some impact on sensitivity after reviewing the final genotype of all confirmed cases. The initial 24-mutation panel would have detected 82.7% of alleles, increasing to 86.1% for the 38-mutation panel, and 90.4% for the final-130 mutation panel. Finally, one case of CFSPID was missed due to a normal repeat IRT at day 21, while a second case was missed due to a normal initial sweat test following an elevated day 21 IRT.

The time to first contact with the CF clinic for follow-up testing based on the screening scenario is presented in Table 2. The median age at referral and first contact with the CF specialist clinic for all true positive cases was 25 days (Range: 10–148 days). Infants with two CFTR mutations were seen by the specialist nine days sooner (Median = 22 days, Range 10–50) than those infants with one mutation who underwent a day 21 IRT (Median = 31 days, Range 12–39). Time to first contact with the CF clinic for the two infants detected by optional sweat testing was significantly longer (72 and 148 days) given the non-urgent pursuit of the testing by these families.

## 4. Discussion

### 4.1. Birth Incidence

Over a nine-year period in British Columbia, 401,977 infants were screened with 76 cases of Cystic Fibrosis detected and an additional 28 infants defined as CFSPID following standard diagnostic guidelines [7]. This yields an incidence for cystic fibrosis of 1/5289 (1/3865 for CF + CFSPID) which is a 30% drop from the 1/3673 incidence for CF in British Columbia estimated by Steinraths et al. (2008) using data from 1993–2005 [10]. Decreasing CF incidence over time has been reported in a number of jurisdictions including a 40% drop in incidence between 1975 and 2005 in Brittany, France, a 43% drop between 1999 and 2006 in Massachusetts, USA, and similar trends reported for other regions of Canada, the UK, Netherlands, and Italy [11,12]. The causes of this decreasing birth incidence are likely multifactorial, with population influxes from regions of historically low CF rates, increased availability of preconception and prenatal testing, and changing case definitions as likely contributors. In fact, the demographic distribution of British Columbia has changed dramatically over the last 25 years with over 30% of the population identifying as a visible minority in 2016 as compared to just 14% in 1991 [13,14]. Over 90% of those identifying as a visible minority have been of Asian ethnic origin, areas with historically low incidence of Cystic Fibrosis [13]. The impact of preconception carrier testing and prenatal genetic testing in BC could not be assessed by the available data.

### 4.2. Program Performance

The addition of the second IRT measurement in the IRT-DNA-IRT algorithm was designed to minimize the number of families required to travel for sweat testing and over the nine-year study period, more than 1000 sweat tests were avoided. The risk with this approach, however, is that a normal second IRT could lead to false negative results. While no CF cases were missed as a result of a falsely normal second IRT (NPV = 100%), two children followed as CFSPID were identified by the optional sweat test arm of the algorithm after a normal second IRT, and a third CFSPID case was missed by screening, and identified through borderline sweat test as part of a clinical evaluation for failure to thrive (NPV = 99.7%).

As designed, this screening algorithm does identify a large number of CF carriers, with 880 apparent carriers identified over the nine-year study period. The reporting of carrier status as part of a newborn screening program creates potential harms for families given a loss of autonomy for the newborn, concerns regarding the residual risk of disease, and heightened perception of the vulnerability of the infant [15]. Our program attempts to minimize these harms through further reducing the residual risk of disease with the second IRT sample, offering an optional sweat test for those with lingering concerns, and providing a dedicated CF liaison nurse to communicate with the families throughout the screening process. The uptake rate for optional sweat testing has been only 13% with most families choosing not to pursue further testing following the normal second IRT result. A retrospective survey of families in 2015 identified a complex set of primary motivators for pursuing the optional sweat test including parental anxiety, a first child, and geographical location [16].

The time to specialist referral can also be significantly impacted by the design of the newborn screening algorithm, particularly where batched DNA testing is utilized or where a second IRT sample is requested. While the median age at first contact with the CF clinic was 25 days for all screen positive infants combined in our cohort, there was a relative nine-day delay for those infants with one CFTR mutation who underwent a second IRT test at day 21. However, for our cohort, the time to first CF clinic visit was comparable to the 0.9 months (~28 days) reported for two other Canadian programs that do not incorporate a day 21 IRT sample [17]. Similar times to specialist referral have been reported in the UK (23 days), and a range of European programs (18–53 days) using a variety of screening strategies [2,18]. A review of US data comparing age at CF specialist referral between states with IRT-DNA and IRT-IRT algorithms showed a median delay of 1.7 weeks for the IRT-IRT states (5.9 vs. 7.7 weeks) but there was significant variability between individual states [19]. US Cystic Fibrosis Foundation consensus guidelines recognize that variation in screening algorithms exists between states but does recommend sweat testing no later than four weeks of age [7]. Current European best practice standards for CF newborn screening recommend that most screen-positive infants be seen by a CF specialists by 35 days of age and no later than 58 days [8]. More than 90% of the infants in our cohort were seen by a CF specialist within this 35-day window.

### 4.3. False Negatives

A total of six CF cases and three individuals with an inconclusive diagnosis (the screen negative equivalent of CFSPID) were missed by the screening program and diagnosed clinically, representing a false negative rate of 7.8% for CF (8.8% for CF+CFSPID). This rate is higher than reported by other screening programs, with false negative rates ranging from 2–8% in the literature, depending on the jurisdiction and approach to screening [20,21,22]. This higher false negative rate may reflect the centralized service for CF in BC and a corresponding likelihood of a clinically ascertained diagnoses being communicated back to the screening program. Importantly, the second IRT and optional sweat testing process did not contribute to the CF false negative rate. Four of the six false negative CF cases (67%) were missed due to low IRT values on the initial screen, none of which were near the 97-percentile daily cutoff (94th, 87th, 79th and 59th percentiles, respectively). The remaining two missed CF cases (33%) had no mutations on the CFTR panel. A similar distribution was reported in California (overall 5% false negative rate) with 50% of cases missed due to low initial IRT, a further 32% missed due to an absence of any mutations on their targeted panel, with the remainder incorrectly identified as carriers when a second mutation was not found on extended sequencing [22]. A lack of sensitivity of IRT for the detection of CF cases remains an issue for programs worldwide. Lowering IRT cutoffs to improve sensitivity would not be a successful strategy based on the levels in our missed cases and those reported by Kharrazi et al., and would significantly increase the amount of mutation analyses required [22]. The use of additional biomarkers such as pancreatitis-associated protein (PAP) in conjunction with IRT can improve specificity but has only minimal impact on sensitivity [23,24,25]. Alternatively, the extension of CFTR molecular testing to include more expansive panels or full gene sequencing cannot address the initial sensitivity issues with IRT and has the unfavorable effect of enriching for CFSPID cases [22,26].

## 5. Conclusions

An IRT-DNA-IRT algorithm, including a day 21 repeat IRT measurement for apparent carriers, was successful in reducing the number of sweat tests required without significantly impacting sensitivity for the detection of CF cases. This algorithm does, however, lead to a slight increase in time to specialist referral for those with one mutation on the CFTR panel, but overall age at first specialist visit is in keeping with both US and European guidelines for the vast majority of infants. As with all CF screening algorithms that rely on IRT as a first-tier screen, the reduced sensitivity of this test remains a concern for false negative screen results.

## Figures and Tables

**Table 1:** Distribution of CF or CFSPID cases in the BC CF Screening Algorithm.

Results	CF		CF + CFSPID	
	Cases Detected	PPV	Cases Detected	PPV
Total Cases (Incidence)	76 (1/5289)		104 (1/3865)	
True Positive	70		95	
Meconium Ileus	3		3	
2 Mutations	57	74%	77	100%
1 Mutation and High IRT2	10	29%	13	38%
1 Mutation and Normal IRT2 (optional sweat test)	0	0%	2 ^1^	1.7%
No Mutations and Top 0.1% IRT1 and High IRT2	0	0%	0	0%
False Negative	6		9	
Normal IRT1	4		5	
No mutations and IRT1 NOT top 0.1%	2		2	
1 Mutation and Normal IRT2 (no sweat)	0		1	
1 Mutation and High IRT2 (Normal Sweat)	0		1	

^1^ Borderline sweat test result.

**Table 2:** Time to first CF clinic contact for screen positive cases.

	Time to First CF Clinic Contact (Days)	
Case Definition (Total)	Median	Range
2 mutations (77)	22	10–50
1 Mutation and High IRT2 (13)	31	12–39
Overall (90)	23	10–50

## Supplementary Materials

The following are available online at https://www.mdpi.com/2409-515X/6/2/46/s1, Table S1: BC Newborn Screening Program CFTR Variant Panel.
